# Postprandial Responses of Serum Cholesterol, Glucose and Plasma Antioxidant Activity, after Intake of an Innovative High Fat Mayonnaise-Based Appetizer, Enhanced with Olive Paste, in Healthy Volunteers

**DOI:** 10.3390/life12091385

**Published:** 2022-09-06

**Authors:** Olga Papagianni, Thomas Loukas, Athanasios Magkoutis, Maria Kagoudi, Dimitrios Skalkos, Dimitrios Kafetzopoulos, Charalampia Dimou, Haralabos C. Karantonis, Antonios E. Koutelidakis

**Affiliations:** 1 Unit of Human Nutrition, Laboratory of Nutrition and Public Health, Department of Food Science and Nutrition, University of the Aegean, 81400 Myrina, Greece; 2 Outpatient Clinic, 81400 Myrina, Greece; 3 Laboratory of Food Chemistry, Department of Chemistry, University of Ioannina, 45110 Ioannina, Greece; 4 Department of Business Administration, University of Macedonia, 54636 Thessaloniki, Greece; 5 Laboratory of Food Chemistry, Biochemistry and Technology, Department of Food Science and Nutrition, University of the Aegean, 81400 Myrina, Greece

**Keywords:** Mediterranean diet, postprandial responses, postprandial bioactivity, bioactive components, olive paste functionality, metabolic biomarkers

## Abstract

Several Mediterranean functional foods and their process by-products may exert a beneficial role on hyperlipidemia, hyperglycemia, and oxidative stress modulation, providing bioactive compounds with functional properties, contributing to possible chronic disease prevention (cardiovascular disease, metabolic syndrome, obesity, diabetes mellitus, etc.). The purpose of the present interventional study was to investigate the postprandial responses of metabolic biomarkers, after the intake of an innovative mayonnaise-based appetizer, enhanced with olive paste, in healthy volunteers. In this cross-over design, randomized and single-blind, interventional–clinical trial, 10 healthy volunteers, aged 20–30 years old, after splitting into the control group and the Mediterranean group, consumed a pasta meal rich in fat and carbohydrates (150 g), containing a mayonnaise-based appetizer or the same appetizer, enhanced with 9% olive paste. After a 1-week washout period, the subjects consumed the meals in reverse. Differences between groups on postprandial responses of total plasma antioxidant capacity according to the FRAP method, serum total cholesterol, glucose, and uric acid levels, were determined before, 30 min, 1.5 h, and 3 h after consumption. The results showed that, in comparison to the control group, consumption of the enhanced appetizer resulted in a significantly decreased total serum cholesterol and glucose levels, and also led to a significant increase in plasma total antioxidant activity, 3 h after consumption (*p* < 0.05). Further investigation with large prospective studies is needed to validate the current results.

## 1. Introduction

In recent years, scientific interest has intensified around the development of innovative functional foods, through the utilization of food processing by-products, which have entered the global market dynamically, in response to the growing consumer needs for new and healthy foods [1]. At the same time, a plethora of in vitro experimental, epidemiological, and clinical studies demonstrate their possible beneficial role, both in maintaining health and in preventing chronic diseases, such as cardiovascular disease, diabetes mellitus, etc., in the context of a balanced diet [2].

The frequent consumption of natural functional foods included in the Mediterranean diet, such as olive oil, wine, mountain tea, etc., has been associated with potential strong antioxidant, anti-inflammatory, antifungal, and other beneficial effects [3]. Due to the bioactive components (e.g., polyphenols, flavonoids, sterols, carotenoids, and unsaturated fatty acids) have shown strong bioactivity, playing an important role in reducing the risk of chronic diseases, as mentioned before [4,5]. Thus, it has been reported that a variety of bioactive, Mediterranean food components and their process by-products, could be used for the enhancement of conventional foods, in order to develop innovative functional foods, since a continuous search for new dietary patterns has been observed [5]. During edible olives packaging, the raw material of inferior quality is discarded from the production line, contributing to the environmental problem, despite their remarkable nutritional value. Under these guidelines, the recovery and reuse of valuable bioactive compounds, through the utilization of natural functional foods of the Mediterranean Diet and food by-products, in the context of maintaining sustainability, seems to be one of the most dynamic prospects.

Additionally, the postprandial state is a recent, interesting field of study through nutritional interventions, as it is a very dynamic period, where numerous metabolic pathways are carried out. The intake of a high-fat and high-carbohydrate meal can lead to impaired, lipemic, and glycemic, postprandial metabolic homeostasis, which has been linked to an increased cardiovascular and diabetic risk [6,7]. On the opposite, previous dietary interventions including functional foods, have shown beneficial effects in regulating postprandial lipemia and glycemia. Supplementing meals, rich in fats and carbohydrates, enhanced with bio-functional ingredients may play a key role in the aforementioned effect [8].

In this context, the objective of this interventional study was to investigate, the postprandial responses of serum total cholesterol and glucose, and of plasma total antioxidant capacity, after a high- carbohydrates and fat meal, containing an innovative mayonnaise-based salad, enhanced with olive paste, produced by the Greek food company “Bioflo”, in healthy volunteers.

## 2. Materials and Methods

### 2.1. Participants

The study protocol was reviewed and approved by the University of the Aegean Ethics Committee (No. 7505, 20 October 2019) and it was conducted in accordance with the Declaration of Helsinki. The study was carried out at the Human Nutrition Unit of the Department of Food Science and Nutrition of the University of the Aegean. All participants were initially informed about the ultimate purpose of the study, the voluntary participation, and the confidentiality of data. A written consent form was obtained from each volunteer before recruitment. After completing a short questionnaire on eating habits and medical history, anthropometric measurements and blood tests followed, in collaboration with external physicians.

The study included healthy volunteers aged between 18–30 years. Exclusion criteria were age <18 and >30 years, taking dietary supplements in the last 2 months, history of chronic disease including type I and II diabetes (hemoglobin A1c-HbA1c > 5%), moderate or heavy smokers (>5 cigarettes/day), abnormal Body Mass Index (BMI) (>25 kg/m^2^) and alcohol overdose (>40 g alcohol/day). In addition, cases with abnormal hematological and biochemical profiles were excluded (cholesterol > 6.8 mM, triglycerides > 2.8 mM, glucose > 6.11 mM). Instructions were given from staff, for the abstention of any medication or dietary supplement during the trial period, as well as from rich in antioxidants foods and alcohol 24 h before participation, while a 12-h fast was requested before the start of the trial period.

### 2.2. Test Meals

The Control meal was consisted of 100 g of cooked penne rigate pasta (Barilla G. E R. Fratelli SPA, Parma, Italy), and 50 g of mayonnaise-based appetizer, supplied by Greek appetizers company, BIOFLO Ltd. (Florina, Greece). The composition of the Mediterranean meal was the same as the Control, except that it contained the enriched with 9% olive paste, mayonnaise-based appetizer, supplied by BIOFLO Ltd. Each whole meal totally weighed 150 g. Dietary composition of the Control and Mediterranean meal is shown in Table 1.

### 2.3. Study Design

The study design included an acute, randomized, and single-blinded, cross-over and two period, interventional trial, separated by a washout period of 1 week. The subjects were randomly divided into two groups of five members, and then assigned to the Control (n = 5) or Mediterranean (n = 5) group, and they crossed over from one arm of the study to the other. Individuals who joined the control group, during each test period, received the control meal, and those who joined the Mediterranean group received the functional meal.

**Figure 1:**
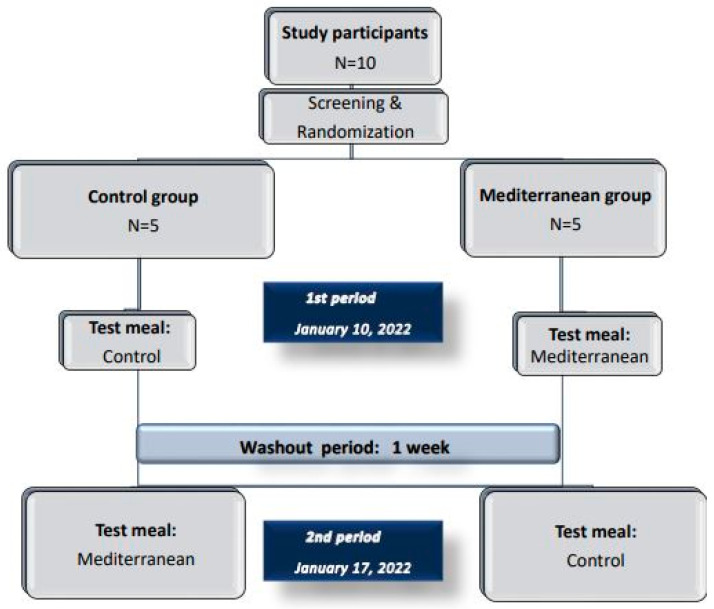
Study design illustration.

The study started on Friday, 10 January 2022. All participants arrived at 9 a.m. at the Nutrition Unit, in an overnight fasting state and in abstinence from dietary supplements, any medication, and food, rich in antioxidants, as requested. The volunteers were asked to complete a self-administrated 24 h recall questionnaire, which recorded all meals consumed in the last 24 h. The study design illustration is shown in Figure 1.

Subsequently, subjects were given a total of 150 g of the meal, including cooked penne rigate (100 g) and mayonnaise-based appetizer (50 g), while a glass of water (250 mL) was available to each participant. In the morning before each visit, the researchers prepared 5 control and 5 Mediterranean meals, due to randomization, in labeled dishes with the subjects’ ID. This procedure was conducted, in order to avoid any kind of volunteers’ bias by knowing the test meal before consumption.

### 2.4. Blood Sampling and Analyses

After an overnight fast, 10 mL of blood sample (baseline) was withdrawn from all participants. The subjects consumed the test meal within 15 min, and postprandial blood samples were taken at 30 min, 1.5 h, and 3 h after completion of the test meal. Blood samples were collected into Ethylenediaminetetraacetic acid (EDTA) and citric acid-treated tubes for plasma separation or heparinized tubes for serum separation. Each subjects’ serum and plasma for each sampling timepoint were immediately separated by centrifugation at 20,000× *g* for 10 min at 4 °C in a tabletop high-speed refrigerated centrifuge (Thermo Scientific ST16R, Thermo Fisher Scientific, Waltham, MA, USA). Serum and plasma were then aliquoted and stored at −40 °C at the research facility (Laboratory Freezer, MRC Laboratory Instruments, Holon, Israel).

Once the study was completed, serum samples were analyzed with an automated biochemical analyzer (COBAS c111, Roche, Basel, Switzerland) for total cholesterol, glucose, and uric acid determination. Total plasma antioxidant capacity (TAC) was evaluated by FRAP assay, as described by Argyri et al. [9,10].

### 2.5. Data Analysis

Statistical analysis was performed by SPSS (SPSS V21.0) and Prism 9 (GraphPad Software Inc., San Diego, CA, USA). Sample calculation power was determined for the primary outcome and venous plasma antioxidant capacity (TAC) using G*Power software version 3.1.9.2 (Universität Düsseldorf). Considering a probability of 95% that the study will detect a treatment difference at a two-sided 0.01 significance level, the sample of 10 individuals allows the detection of a difference of 0.2 mmol TAC/L between the control group and the intervention group, calculated from the expected SD = 0.2 between the differences of the meal groups. Results are presented as mean ± standard deviation (SD) and statistical significance was accepted at *p* ≤ 0.05. Normality of distributions was assessed performing the Kolmogorov–Smirnov test. The main participants’ characteristics were analyzed by descriptive statistics. Serum cholesterol, glucose, uric acid, and plasma total antioxidant capacity (TAC) were analyzed via repeated ANOVA measures, with Geisser–Greenhouse correction. Post-hoc tests were performed via the Bonferroni test. The Wilcoxon sign-rank test was performed on changes in the clinical characteristics from baseline to postprandial responses (within-group variation).

Sample calculation power was determined for the primary outcome, venous plasma antioxidant capacity (TAC) using Statmate version 2.0 (GraphPad Software Inc., San Diego, CA, USA). Taking an α = 00.01, a sample size of 10 subjects allows for the detection of a difference of 0.21 mmol TAC/L between groups, calculated from an expected SD of between-meal group differences of 0.21 mmol/L.

## 3. Results

### 3.1. Study Population Characteristics

The initial study population comprised 10 healthy volunteers, 5 men, and 5 women who fulfilled the selection criteria. All initial participants completed the study. Table 2 shows the baseline characteristics of the study population.

### 3.2. Values for Age, Weight, Height and BMI Represents Mean ± SD

Analysis of the food frequency questionnaires showed that the majority of subjects were consuming fruits 3–4 times per week, vegetables 1–2 times per week, and starch-rich foods every day, while they declared that they include olive oil in their diet, but not olive products. Two participants were occasional light smokers (1–3 cigarettes per day).

### 3.3. Baseline and Postprandial Responses of Test Meals on Metabolic Biomarkers

Table 3 shows baseline and postprandial changes data for glycemic, lipemic profile, and plasma antioxidant status in both control and Mediterranean groups.

Baseline values for some biomarkers show deviations, due to the randomization process and the high variation of function and metabolism of each individual human organism. However, the importance of the findings is focused on the different postprandial responses of the two test meals.

**Figure 2:**
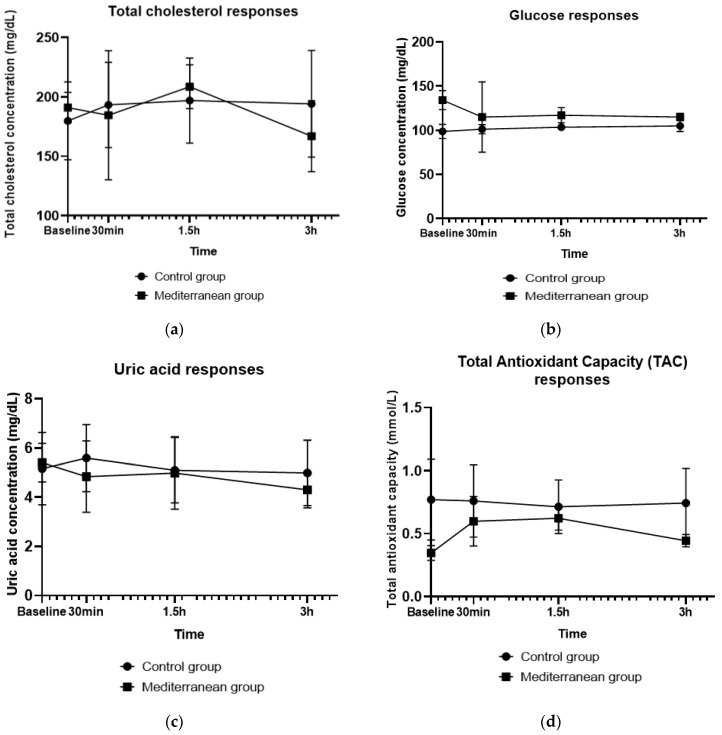
Postprandial responses on test meals of (**a**) Serum total cholesterol; (**b**) Serum glucose; (**c**) Serum uric acid; (**d**) Plasma Total Antioxidant Capacity (TAC).

A significant group x time interaction (*p* = 0.014) was found for serum total cholesterol. As shown in Figure 2a, postprandial total cholesterol following the Mediterranean meal, containing the enhanced olive paste, appetizer, significantly reduced in the first 30 min after meal consumption (*p* = 0.045), following a significant raise until 1.5 h (*p* = 0.041), and finally 3 h after meal intake reduced significantly (*p* = 0.028), reaching lower levels than baseline. Contrariwise, the total cholesterol of the control group, increased significantly during the first 30 min (*p* = 0.046), and then there were no significant changes until endpoint (*p* > 0.05).

As shown in Figure 2b, the meal containing the enhanced with olive paste, a mayonnaise-based appetizer, led to significantly reduced serum glucose levels at 30 min after meal intake (*p*= 0.043), and no significant changes were observed until the endpoint, while in the control group no significant changes were found at any time point. However, no one group x time interaction was detected for this metabolic biomarker.

Figure 2c shows postprandial responses of test meals on serum uric acid concentrations. A group x time interaction was detected for uric acid concentrations (*p* = 0.006). Uric acid was significantly reduced at 30 min after Mediterranean meal consumption (*p* = 0.034), when nonsignificant responses on control meal (*p* > 0.05), were found for postprandial uric acid.

A significant group x time outcome (*p* = 0.01) and a group effect (*p* = 0.027) was, also, observed for plasma Total Antioxidant Capacity (TAC). Particularly, as presented in Figure 2d, total plasma antioxidant activity, significantly increased (*p* = 0.011) in the first 30 min after Mediterranean meal consumption, but it significantly decreased at the last 1.5 h (*p* = 0.011). Though, at the endpoint plasma, TAC of the Mediterranean group was significantly reduced (*p* = 0.034), compared with baseline values. Plasma TAC values did not change significantly at any timepoint (*p* > 0.05), following the mayonnaise-based, control meal.

## 4. Discussion

High consumption of olive products, as a defining feature of the Mediterranean diet, has extensively been associated with a reduced risk of metabolic disease, due to the presence of polyphenols and other bioactive compounds [11]. Recent studies suggest the possible utilization of food by-products, such as olive milling wastes, in order to develop value-added foods, with high nutritional worth, enhanced in bioactive compounds [12]. The antihyperglycemic, hypolipidemic and antihypertensive effects of olive products’ polyphenols have been mentioned in numerous, human interventional studies, both in healthy volunteers and metabolic patients [11]. This was the first nutritional intervention to investigate the acute effect of a mayonnaise-based appetizer, enhanced with olive paste, on postprandial serum cholesterol, glucose, and plasma total antioxidant capacity (TAC) levels.

Previous studies indicate that the improvement of postprandial plasma oxidative status, in combination with excessive lipemic and glycemic response suppression, could be achieved by supplementation of high-fat and carbohydrates meals, with plant bioactive compounds [13].

In the present study, acute consumption of the mayonnaise-based appetizer, enhanced with olive paste bioactives, led to statistically significant decreased total cholesterol levels, 3 h after meal intake. To the best of our knowledge, the phenolic supplementation of rich fats, and meals, does not significantly affect total cholesterol levels in the postprandial state [14]. However, the intake of oleic acid contained in the olive paste, chosen for fat meal enhancement, has been reported to lead to lower levels of total cholesterol and LDL cholesterol, and this could explain the reported significant difference [15]. Recently, an interesting correlation between changes in circulating miR-17-5p levels and changes in total cholesterol and oxLDL, after the ingestion of polyphenol-enriched, olive oil. Daimiel et al. suggested that postprandial modulation of circulating microRNA levels, associated with olive products’ intake, is a potential mechanism for cardiovascular protection [16].

The second finding of this nutritional intervention was the significant glucose levels’ reduction, 30 min after the olive paste enriched, high- carbohydrate meal. This effect may be explained due to the direct suppression of proteins involved in the intestinal transport of dietary carbohydrates, attributed to olive polyphenols [17]. Carnevale et al., in a nutritional intervention, found that a meal supplementation with extra virgin olive oil, resulted in a 20% reduction in postprandial glucose and a 40% increase in insulin, focusing their results on incretins’ effect, which act as post-prandial glycemia regulators, by up-regulating insulin secretion [18].

The last significant finding of this clinical study was the increased plasma TAC of the volunteers who consumed the Mediterranean appetizer, 3 h after the meal intake. The polyphenolic metabolites (tyrosol, hydroxytyrosol, oleuropein), derived from olive paste, may have been absorbed and released into the circulation within the first half hour after meal ingestion, and may have been gradually excreted [14]. In the present study, plasma values were found to be significantly higher than baseline, after consuming the Mediterranean meal, which was not observed after taking the control meal. This could be explained by the transfer of Mediterranean mela’ bioctives or its metabolites on circulation after their absorption and metabolism. Similar findings have been reported in our previous study, where plasma antioxidant activity was found to be significantly increased, 3 h after consuming functional cookies, fortified with olive paste [14].

The effect of meals on the postprandial uric acid response was investigated, as uric acid is an endogenous antioxidant and may contribute to the increase of total plasma antioxidant capacity [8]. Nevertheless, we saw a significant decrease in its concentrations, in the last 1.5 h, in the group that consumed the Mediterranean meal. These findings strengthen our claims for the beneficial effect of polyphenols on postprandial, blood antioxidant status improvement.

Some limitations of the present study should be underlined. Although the adequacy of the sample size of volunteers participating in the study was statistically calculated, the sample size may have influenced the lack of statistical significance for the remaining biomarkers tested. Further studies with a higher number of subjects, both healthy and with high cardiometabolic risk, should be conducted, in order to accurately investigate if the enhanced with olive paste, a mayonnaise-based appetizer, may have more pronounced effects in the postprandial state. In addition, the specific phenolic compounds of the Mediterranean meal did not calculate, but only the total phenolics content was determined. Finally, a major limitation is that herein we studied the effect of acute consumption of enhanced, high-fat appetizer, in healthy volunteers as a postprandial preventive nutritional parameter; however, higher positive effects may be observed by long-term consumption, so future, long-term interventional studies could exact results of increased clinical interest.

## 5. Conclusions

The findings of the present study highlighted the possible beneficial effect of a mayonnaise-based, rich in fat, appetizer enrichment, with bioactive compounds, derived from olive paste, on postprandial biomarkers of lipemia, glycemia and oxidative stress, blamed for increased cardiovascular and metabolic risk.

Concluding, in the context of the low adherence of modern consumers to the Mediterranean diet pattern, the enhancement of conventional foods with bioactives from food by-products and natural functional foods, is one of the most dynamic approaches for eating habits improvement and chronic disease prevention. Nevertheless, these findings are an indication of the possible, protective effect of polyphenol-enriched, innovative food consumption, and it is necessary to further investigate their postprandial effect on more lipidemia, glycemia, and oxidative stress biomarkers, in order to draw safer conclusions.

## Figures and Tables

**Table 1:** Dietary composition of the tested meals (150 g).

Nutrients	Mediterranean	Control
Energy (kcal)	275.21	280.42
Carbohydrates (g)	20.6	17.96
Fat, total (g)	18.71	16.49
Protein (g/kg)	5.5	3.52
Saturated fat (g)	4.01	10.96
Unsaturated fat (g)	14.7	5.54
Cholesterol (mg)	12.09	14.1
Dietary fiber, total (g)	1.34	1.12
Sugar, total (g)	1.4	1.35
Total Phenolics (mg)	4.7	0

**Table 2:** Study Participants’ baseline characteristics.

Participants’ Baseline Characteristics	
Participants (number)	10
Men (number)	5
Women (number)	5
Dietary supplementation (number of participants)	0
Physical activity medium or high (number of participants)	8
Age (years)	24.7 ± 1.4
Weight (kg)	67.2 ± 7.2
Height (cm)	172 ± 11.6
BMI	22.7 ± 0.3

**Table 3:** Baseline clinical characteristics and postprandial responses of test meals on metabolic biomarkers.

		Variable	Total Cholesterol (mg/dL) Mean (SD)	Glucose (mg/dL) Mean (SD)	Uric Acid (mg/dL) Mean (SD)	Total Antioxidant Capacity [TAC] (mmol FeSO_4_/L) Mean (SD)
Baseline values		Control group	179.87 (32.86)	99.25 (7.51)	5.16 (1.47)	0.77 (0.032)
		Mediterranean group	191 (12.82)	134.12 (10.78)	5.4 (0.78)	0.34 (0.056)
Postprandial changes	30 min	Control group	13.46 (9.51)	2.25 (1.96)	0.43 (0.3)	−0.01 (0.007)
		Mediterranean group	−33.5 (4.5)	−19.2 (13.5)	−0.56 (0.39)	−0.17 (0.12)
	1.5 h	Control group	3.7 (2.59)	2 (1.41)	−0.49 (0.34)	−0.05 (0.00)
		Mediterranean group	50.64 (16.97)	2 (1.41)	0.14 (0.1)	0.03 (0.00)
	3 h	Control group	−2.86 (2.02)	1.87 (1.32)	−0.11 (0.07)	0.03 (0.01)
		Mediterranean group	−45.14 (29.4)	−2 (1.41)	−0.68 (0.48)	−0.18 (0.01)

## Data Availability

The data presented in this study are available within this article.
